# Evaluation of the Mutational Preferences Throughout the Whole Genome of the Identified Variants of the SARS-CoV-2 Virus Isolates in Bangladesh

**DOI:** 10.3390/ijms26136118

**Published:** 2025-06-25

**Authors:** Laila Anjuman Banu, Nahid Azmin, Mahmud Hossain, Nurun Nahar Nila, Sharadindu Kanti Sinha, Zahid Hassan

**Affiliations:** 1 Department of Anatomy, Bangladesh Medical University, Dhaka 1000, Bangladesh; 2 Genetics and Molecular Biology Laboratory, Bangladesh Medical University, Dhaka 1000, Bangladesh; 3 Department of Anatomy, Shahabuddin Medical College, Dhaka 1212, Bangladesh; nahidazmin@gmail.com; 4 Laboratory of Neuroscience and Neurogenetics, Department of Biochemistry and Molecular Biology, University of Dhaka, Dhaka 1000, Bangladesh; mahmudbio1480@du.ac.bd (M.H.); nurunnahar-2015517630@bmb.du.ac.bd (N.N.N.); 5 Department of Pharmacology, Bangladesh Medical University, Dhaka 1000, Bangladesh; sharadindu@bsmmu.edu.bd; 6 Department of Physiology and Molecular Biology, Bangladesh University of Health Sciences, Dhaka 1216, Bangladesh; mzhassan@buhs.ac.bd

**Keywords:** SARS-CoV-2, delta, omicron, mutation, substitution, deletion, nucleotide, amino acid, phylogeny, Bangladesh

## Abstract

The study aimed to identify the variants of SARS-CoV-2 (Severe Acute Respiratory Syndrome related coronavirus-2) virus isolates within the window of March 2021 to February 2022 in Bangladesh and investigate their comparative mutational profiles, preferences and phylogenetics. After the collection of the sample specimen and RNA extraction, the genome was sequenced using Illumina COVID Seq, and NGS data analysis was performed in DRAGEN COVID Lineage software (version 3.5.9). Among the 96 virus isolates, 24 (25%) were from Delta (clade 21A (*n* = 21) and 21J (*n* = 3)) and 72 (75%) were from Omicron (clade 20A (*n* = 6) and 20B (*n* = 66)). In Omicron and Delta, substitutions were much higher than deletions and insertions. High-frequency nucleotide change patterns were similar (for C > T, and A > G) in both of the variants, but different in some (i.e., G > T, G > A). Preferences for specific amino acids over the other amino acids in substitutions and deletions were observed to vary in different proteins of these variants. Phylogenetic analysis showed that the most ancestral variants were from clade 21A and clade 20A, and then the other variants emerged. The study demonstrates noteworthy variations of Omicron and Delta in mutational pattern and preferences for amino acids and protein, and further study on their biological functional impact might unveil the reason behind their mutational strategies and behavioral changes.

## 1. Introduction

Coronaviruses are highly pathogenic, and are the largest group of viruses [1] which are the causative agents of severe respiratory diseases, extra-pulmonary disease conditions, and, in critical cases, death in humans [2]. Two pandemics broke out in 2002 and 2012, with the Severe Acute Respiratory Syndrome (SARS)-related coronavirus (SARS-CoV), and Middle East Respiratory Syndrome (MERS)/related coronavirus (MERS-CoV), respectively [3]. After these two, the third wave of devastating pandemic emerged as the Coronavirus disease 2019 (COVID-19), in December of 2019, which posed a great threat not only to immunocompromised individuals but also to healthy adults [4]. As of January 2025, SARS-CoV-2 has infected over 777 million people and caused the deaths of around 7.1 million people [5] (last accessed on 10 January 2025).

SARS-CoV-2 is an enveloped, positive-sense single-stranded RNA virus. The genomic RNA is approximately 30 kb in length and it contains two untranslated regions at the two ends and 14 ORFs (Open Reading Frames) that encode 16 non-structural proteins (NSPs), structural proteins named spike protein (S), membrane protein (M), nucleocapsid protein (N), and envelope protein (E), and a set of accessory proteins including ORF3a, ORF3b, ORF6, ORF7a, ORF7b, ORF8, and ORF9b [6]. Apart from the crucial role of NSPs in the formation of RNA-dependent RNA polymerase (RdRp) holoenzyme, formation of the replication organelle, and synthesis of viral proteins, they play an important role in manifesting abnormal immune response in a host, as well as in immune evasion [6,7]. The structural protein, N, plays a vital role in shielding the viral RNA genome from the host cytoplasmic immune surveillance by antagonizing the IFN-β (Interferon-beta) response [8]. The spike protein is essential for entry into the host cell [9], and this protein comprises two subunits—the S1 subunit has a Receptor Binding Domain (RBD) that attaches to and recognizes the receptor protein (i.e., angiotensin-converting enzyme 2 (ACE2)) of the host cell and the S2 subunit initiates membrane fusion [10], which is regulated by the innate immune factors of the host [9,11]. Since the beginning of the COVID-19 pandemic, numerous mutations of SARS-CoV-2 have been identified. Periodic viral genomic sequencing helps to detect new genetic variants circulating in communities [12]. An updated version of the SARS-CoV-2 phylogenetic tree is shared on GISAID platform (Global Initiative on Sharing Avian Influenza Data). A variant is recognized as a Variant of Concern (VOC) or Variant of Interest (VOI) by the World Health Organization (WHO) [12]. Numerous substitution mutations were reported in the Heptapeptide Repeat and Fusion peptide domains of the S protein which were implicated to impact biochemical properties and potentially increase viral pathogenicity [13]. The accessory proteins are believed to be virulence factors that contribute to several pathogenesis pathways and immune evasion, i.e., ORF3a, ORF7a, and ORF7b, block IF-α signaling and disrupt the phosphorylation of STAT1/2, and ORF8 represses IFNβ signaling [14]. Furthermore, the introduction of vaccines against SARS-CoV-2 changed the course of the pandemic [12]. In fact, the recent development of vaccines was considered a powerful measure to save lives and minimize the impact on health, social systems, and global economics [15]. It is well known that SARS-CoV-2 genome mutations influence the efficacy of the immune response induced by vaccination [12]. 

A comprehensive analysis of the mutation patterns and preferences in different variants of SARS-CoV-2 virus is necessary to understand their evolution, viral behaviour, and survival strategies in Bangladesh [16,17,18]. In this study, 96 SARS-CoV-2 variant isolates were sequenced, identified, and their mutation patterns and preferences were evaluated and compared from different angles, along with the whole genome phylogenetic analysis, which may add some information to the global dataset of SARS-CoV-2 from Bangladesh.

## 2. Results

### 2.1. Socio-Demographic Characteristics of the COVID-19-Positive Patients

In this study, the frequency of female COVID-19-positive patients (51.04%) was higher than that of the males (48.96%, Table 1). The distribution of males and females varied in different divisions (Supplementary Figure S1). The frequencies of patients with asthma, diabetes mellitus, hypertension, cardiovascular disease, chronic kidney disease, and other comorbidities were 9, 31, 25, 3, 9, and 11, respectively, whereas 8 of the COVID-19 patients had no comorbidities (Table 1).

However, 71 of the patients were vaccinated, whereas 25 were not (Table 1). The frequency of the vaccinated and non-vaccinated patients in different divisions is presented in Supplementary Figure S2. Moreover, 12 patients had a history of long-distance travelling and 81 did not (Table 1). Additionally, 31 patients had a family history of COVID-19 infection and 29 did not, while 36 (37.50%) of them could not confirm the information (Table 1). Regarding reinfection, 18 patients were reinfected with COVID-19, whereas 78 were not (Table 1). The Supplementary Figure S3 shows the status of reinfected and non-reinfected patients in different divisions; moreover, the visual representation of their reinfection and vaccination status is presented in the Supplementary Figure S4, which shows that 15 of the 96 patients were reinfected with SARS-CoV-2, even though they were vaccinated.

### 2.2. Identified Variants, Clades and Lineages

The frequencies of the identified Delta and Omicron variants were 24 and 72, respectively (Table 2). This study identified two clades of Delta, i.e., 21A as well as 21J, and two clades of Omicron, i.e., 20A as well as 20B. Clades 21A and 21J were identified in 21 and 3 of the patients. The frequencies of clades 20A and 20B were 6 and 66, respectively (Table 2).

**Figure 1:**
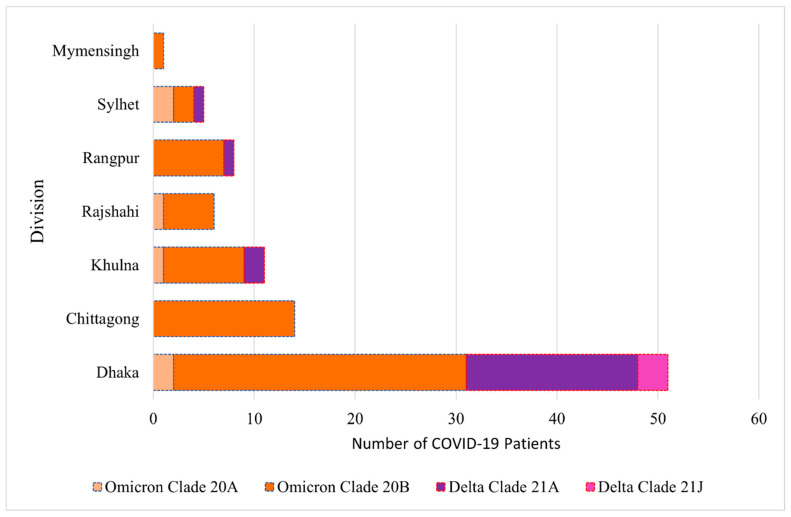
Distribution of the clades of the Delta and Omicron variants in different divisions of Bangladesh.

Clade 20B was the predominant clade in all the divisions (Figure 1). The second most high-frequency clade was identified to be 21A. Omicron 20A clade was observed in all the divisions except Chittagong, Rangpur, and Mymensingh, whereas a less frequent Delta 21J clade was reported only in the Dhaka Division (Figure 1). In this study, the Delta variants were from eight lineages (AY.131, AY.122, AY.116, AY.121, AY.123, AY.127, AY.4.4, and B.1.617.2) and the Omicron variants were from five lineages (BA.2, BA.1, BA.1.1, B.1, and B.1.1.529, Supplementary Table S1).

### 2.3. Comparison of the Total Number of Mutations in Four Clades

Genome-wide mutation analysis revealed that the frequencies of mutation types in all four clades were almost the same, following the order of nucleotide substitution, amino acid substitution, amino acid deletion, and insertions (Supplementary Figure S5). From Supplementary Figure S5, it is obvious that compared to clades 21A and 21J, the frequencies of deletions were higher in clades 20A and 20B.

### 2.4. Analysis of Nucleotide Substitutions at Variant and Clade Level

Table 3 presents the nucleotide substitutions that were observed in more than 10% of the 96 SARS-CoV-2 viruses, and the rest of the mutations are shown in Supplementary Table S2. Nineteen substitutions (C10029T, C27752T, T26767C, T27638C, A23403G, G15451A, G210T, C241T, C25469T, C14408T, G28881T, G28916T, C27874T, C16466T, C19220T, G4181T, C6402T, A11201G, A11332G) were present in all of the twenty-four Delta variants (Table 3). However, 7 substitutions (C3037T, C14408T, A18163G, C23525T, T23599G, C23604A, A24424) were noted in all of the 72 variants of Omicron.

**Figure 2:**
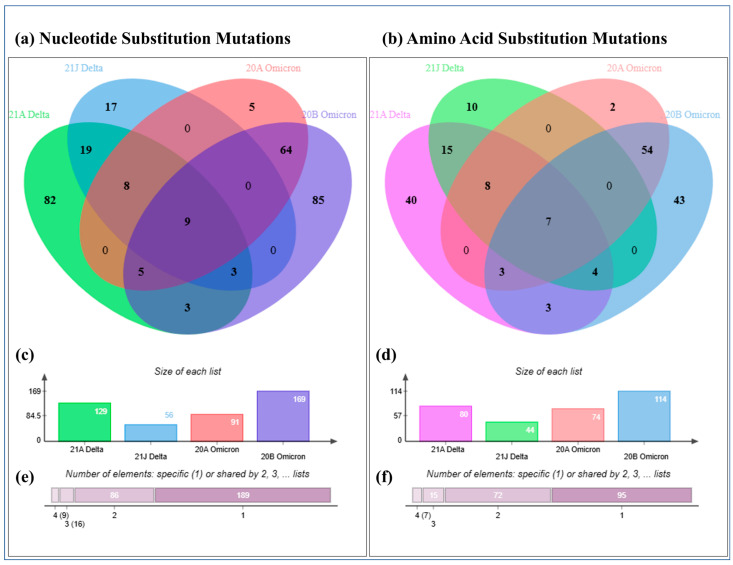
Venn chart of the nucleotide substitutions and amino acid substitutions in Delta and Omicron. Number of distinctive unique and common nucleotide (**a**) and amino acid (**b**) substitutions in four different clades. Bar chart of the total number of distinctive nucleotide (**c**) and amino acid (**d**) substitutions in four different clades. Total number of distinctive nucleotide (**e**) and amino acid (**f**) substitutions that were unique and commonly present in four different clades.

Figure 2a shows that 20B and 21A harbour the highest number of unique substitutions. The distinctive, unique mutations are provided in the supplementary datasheet named “Unique Mutations”. Moreover, nine distinctive substitutions were observed in the four clades (C241T, C10029T, C3037T, C14408T, A23403G, C22995A, C21846T, C27874T, and C28054G (Figure 2a, Supplementary Table S3)). The frequencies of distinctive substitutions in 20B, 21A, 20A, and 21J were 169, 129, 91, and 56, respectively (Figure 2c).

### 2.5. Evaluation of Nucleotide Base Change Pattern in Delta and Omicron Variants

The order of high-frequency nucleotide base change in Delta was C > T (39.26%), G > T (23.01%), A > G (12.43%), T > C (7.20%), C > G (6.98%), and G > A (4.47%), whereas in Omicron the order was C > T (35.59%), G > A (14.36%), A > G (11.17%), C > A (8.96%), A > T (5.58%), T > G (5.37%), T > C (5.27%), and A > C (4.43%), and so on (Table 4). Both in Omicron and Delta, the first preference was C > T pyrimidine change, and G > T was in second place in Delta, whereas G > A was in second place in Omicron and A > G was in third place in Delta and Omicron.

In both of the variants, the percentages of transition mutations were almost 2-fold higher than the transversion mutations (Supplementary Table S4), and transition frequency was lower in Delta (63.36%) than in Omicron (66.39%). In contrast, transversion frequency was higher in Delta (36.64%) and lower in Omicron (33.61%).

### 2.6. Analysis of Amino Acid Substitutions at Variant and Clade Level

The nucleotide substitutions that result in amino acid substitutions in protein products are presented in Table 5. In addition, Supplementary Table S5 shows the amino acid substitutions that were mainly observed in less than 4% of the Omicron variant. Fourteen substitutions (ORF1a:A1306S, ORF1a:T3255I, ORF1a:T3646A, ORF1a:P2046L, ORF1b:A1918V, ORF1b:G662S, ORF1b:P1000L, S:D614G, ORF3a:S26L, M:I82T, ORF7a:T120I, ORF7b:T40I, N:G215C, and N:R203M) were present in all of the Delta variants (Table 5).

In Delta, other high-frequency substitutions were ORF1a:V2930L, S:T19R and S:P681R. Six substitutions, ORF1b:I1566V, ORF1b:P314L, S:P681H, S:Q954H, S:H655Y, and S:N679K, were observed in all of the Omicron variants, and other high-frequency mutations were ORF1a:T3255I, ORF1a:P3395H, S:G142D, S:D614G, E:T9I, M:A63T, ORF9b:E27V, ORF9b:P10S, N:P13L, N:G204R, and N:R203K (Table 5). Contrasting frequency in Delta and Omicron was observed for several substitutions, including ORF1a:P2046L, ORF1a:T3646A, ORF1b:A1918V, ORF1b:G662S, ORF1b:P314L, S:A67V, S:E156G, S:G142D, S:G446V, S:L452R, S:N969K, S:T19R, ORF3a:S26L, M:I82T, ORF7b:T40I, ORF8:S54*, and N:M1X, where all of them had *p* values of <0.05 (Supplementary Table S6). Other mutations having statistically significant difference in frequencies in Delta and Omicron variants were ORF1a:I880V and S:N501T (Supplementary Table S6).

Figure 2b depicts the fact that the numbers of unique amino acid substitutions in clade 21A, 21J, 20A, and 20B were 40, 10, 2 and 43, respectively, and, in total, 95 unique substitution mutations were observed (Figure 2f). Seven mutations (ORF1a:T3255I, S:D614G, ORF7b:T40I, ORF8:S54*, S:T478K, S:T95I, and ORF1b:P314L) were observed in the four clades (Supplementary Table S7, Figure 2b). Moreover, Figure 2d shows that clades 20B, 21A, 20A, and 21J contained 114, 80, 74, and 44 distinctive amino acid substitutions, respectively.

### 2.7. Investigation of Deletion Mutations at Variant and Clade Level

The pattern of deletion mutations was analysed at the clade level (Supplementary Table S8), which showed that the 24 variants of Delta had five deletion patterns and 20 variants of clade 21A followed a single pattern of deletion. The 6 variants of clade 20A had 5 deletion patterns, and 66 variants of clade 20B had 6 patterns of deletion; among these 6 patterns, 3 of them were followed by the majority, i.e., 27, 21, and 14 variants of clade 20B.

Edward’s Venn diagram in Supplementary Figure S6a indicates that one amino acid deletion (S:Y144-) was found in all of the four clades (Supplementary Table S9). Amino acid deletions that were observed to be common in two or more clades are provided in Supplementary Table S9. Clade 20A and 21A contained no unique deletions, whereas clade 21J and 20B contained one and five, respectively (Supplementary Figure S6a). The bar chart of Supplementary Figure S6b shows that, in total, clades 20B and 20A had a higher number of distinctive deletions than 21A and 21J.

### 2.8. Comparison of Amino Acid Substitutions and Deletions in Proteins

**Figure 3:**
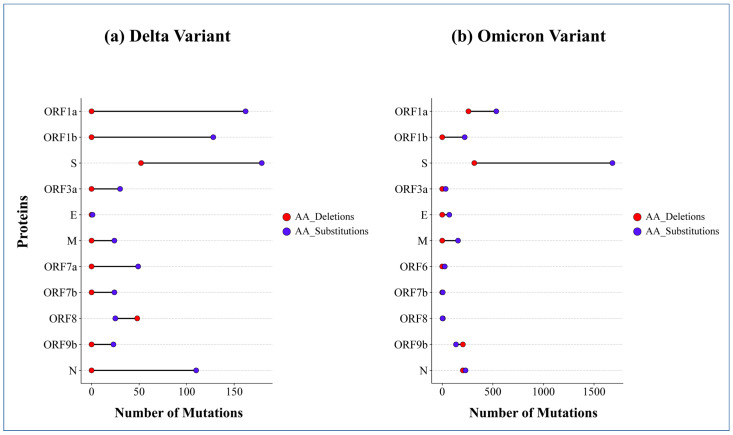
Dumbbell chart of the frequency of amino acid (AA) deletions and substitutions in different structural and non-structural proteins of the SARS-CoV-2 viral genomes of Delta (**a**) and Omicron (**b**) variants. Here, ORF 1a, 1b, 3a, 6, 7a, 7b, 8, and 9b = Open Reading Frame 1a, 1b, 3a, 6, 7a, 7b, 8, and 9b protein, S = Spike protein, E = Envelope protein, M = Membrane protein, N = Nucleocapsid protein.

In Delta, no deletion was observed in ORF1a, ORF1b, ORF3a, E, M, ORF7a, ORF7b, ORF9b, and N protein; rather, substitutions were reported in these proteins (Figure 3a). For deletion, Delta preferred the S and ORF8 protein. Comparison of amino acid deletions and substitutions in the S and ORF8 protein depicts the fact that Delta preferred more substitutions in the S protein than deletions, and it preferred more deletions in the ORF8 protein than substitutions.

In Omicron, the S protein had a notably high frequency of substitutions, and was followed by ORF1a, ORF1b, M, and N protein (Figure 3b). In Omicron, the number of deletions is high in the S, ORF1a, ORF9b, and N proteins. Compared to other proteins, a low frequency of substitutions was observed in ORF3a, E, ORF6, ORF7b, ORF8, and ORF9b proteins of Omicron, and among them deletions were observed in ORF8, and ORF9b, but not in ORF3a, E, M, ORF6, and ORF7b (Figure 3b).

### 2.9. Preferred Substituted Amino Acids of Substitution Mutations

**Figure 4:**
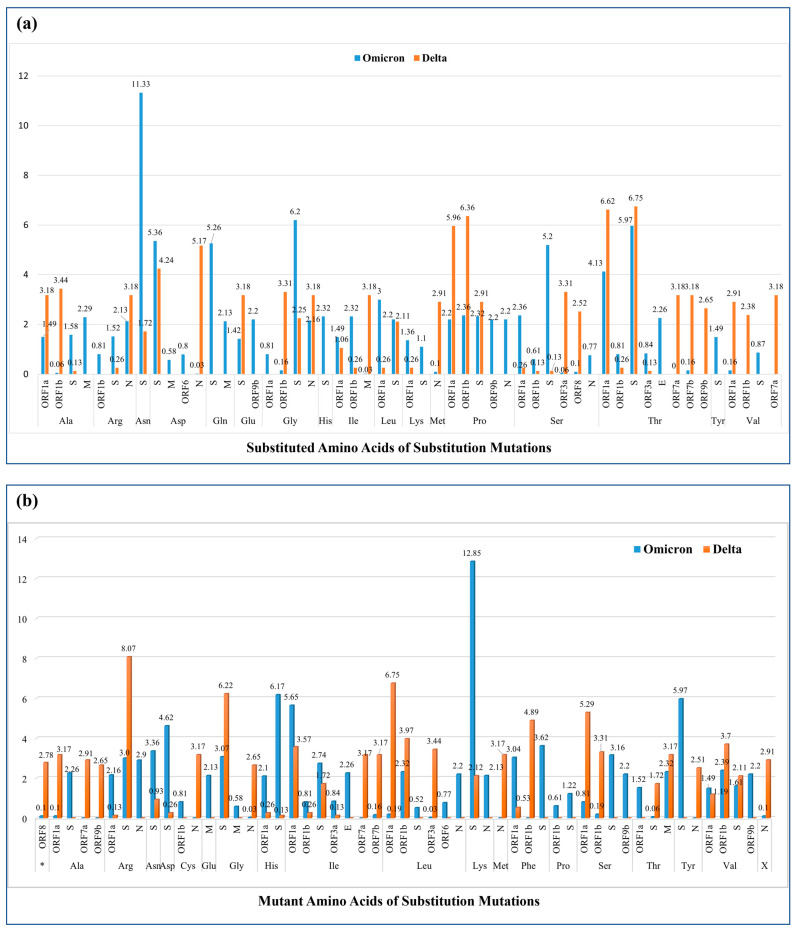
Percent column chart of the substituted (**a**) and mutant (**b**) amino acids in different proteins of the Omicron and Delta variants. Here * indicates a stop codon that does not code for any amino acid.

Omicron substituted the wild-type amino acids—Asn, Gly, Thr, Asp, Ser, and Gln of the spike protein at a high frequency, and His, Lys, Tyr and Val at a low frequency, whereas in Delta, the order of preferences was Thr, Asp, Glu, Pro, Gly, and Leu (Figure 4a). The preference for Asn and Ser in the S protein of Omicron contrasts with that in Delta. In ORF1a protein, Delta and Omicron preferred Ala, Val, Leu and Ser in a contrasting manner. Regarding ORF1b protein, Delta and Omicron showed contrasting preference for Ala, Gly, Val, and Ile. In the N protein, contrasting preference was observed for Asp, Met, Pro, and Ser in Deta and Omicron. In the M protein, differential preference was also observed for Ala, Gln, and Ile in Omicron and Delta (Figure 4a). Contrasting preference for Thr, Glu, and Pro was also observed in the ORF9b protein of Delta and Omicron. In ORF7a protein, the substitution of Thr and Val was observed in Delta, but not in Omicron. Contrasting preference was also observed in ORF7b, E, ORF3a, and ORF8 protein for Thr, Thr, Ser, and Ser, respectively, in Delta and Omicron.

### 2.10. Preferred Mutant Amino Acids of Substitution Mutations

Taking all the mutant amino acids’ number of occurrences in a variant as a whole, the percentages of the mutant amino acids in different proteins were calculated (Figure 4b). In the S protein, preference for mutant Lys, Arg, Gly was observed in Omicron and Delta and they showed a differential preference for His, Tyr, Phe, Asp, Asn, Ser, and Ala. In ORF1a protein of Delta and Omicron, a high preference for Ile was observed, whereas Delta and Omicron showed a differential preference for mutant Leu, Ser, Ala, Phe, His, and Arg. In ORF1b protein, the percentages of mutant Phe, Leu, Ser, and Val in Delta vs. Omicron were 4.89% vs. 0%, 3.97% vs. 2.32%, 3.31% vs. 0.19%, and 3.70% vs. 2.39%, in this order. Regarding ORF8 protein, the percentage of mutant stop codon was higher in Delta than in Omicron. For substitution in N protein, Omicron and Delta showed differential preference for Arg, Leu, Lys, Cys, Met, Gly, and Tyr (Figure 4b). Preferences for other mutant amino acids in other proteins are shown in the same figure.

### 2.11. Analysis of Deleted Amino Acids in Delta and Omicron Variants

**Figure 5:**
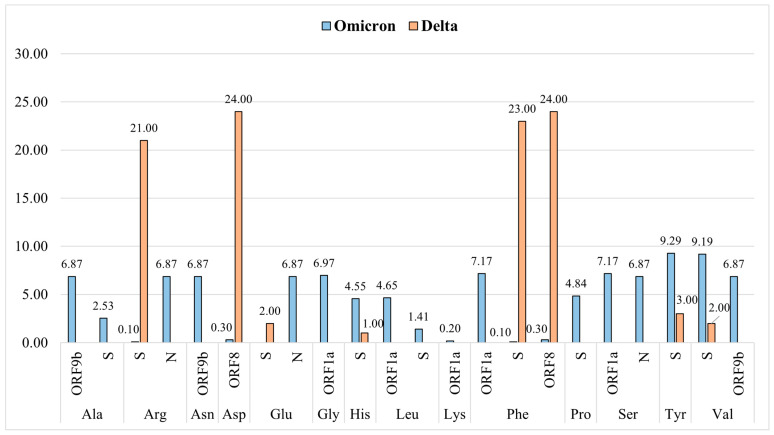
Percent column chart of the deleted amino acids in the Omicron and Delta variants.

In Delta, deletion was observed merely in the S, as well as ORF8 protein, and for deletion in ORF8 protein, Delta exclusively selected Asp and Phe residues, whereas for deletion in S protein, Delta primarily selected Phe, as well as Arg residues, and it preferred Tyr, Glu, Val, as well as His residues at a low frequency (Figure 5).

On the other hand, in the S protein of Omicron, Tyr, Val, and Pro were deleted the most. Moreover, deletion of His, Ala, and Leu was also observed in the S protein of Omicron. In the ORF1a protein of Omicron, Phe, Ser, Gly, and Leu were deleted and for deletion in ORF9b protein, Ala, Asn, and Val were selected by Omicron. Moreover, for deletion, Omicron selected Arg, Glu, and Ser residues of the N protein.

### 2.12. Insertion Mutations in Delta and Omicron Variants

In a variant of clade 21A, one insertion mutation (2902:GTGTTGTGGCAG) of 12bp length was observed in ORF1a protein and a 9bp insertion (22206:GCCAGAAGA) was reported in the spike protein of 11 samples of clade 20B (Table 6). These two insertion mutations are frame preserving and insert “VLWQ” amino acids at the N-terminal domain of nsp3a and “EPE” amino acids at the N-terminal domain of the S1 subunit of the spike glycoprotein, respectively. Moreover, in a sample of clade 20A, two insertion mutations, 75:AAAC and 76:AAA, of 4bp and 3bp in length, respectively, were observed in the 5′ leader sequence, which also did not result in frameshift mutations.

### 2.13. Phylogenetic Analysis of the Whole Genome of SARS-CoV-2 Virus

**Figure 6:**
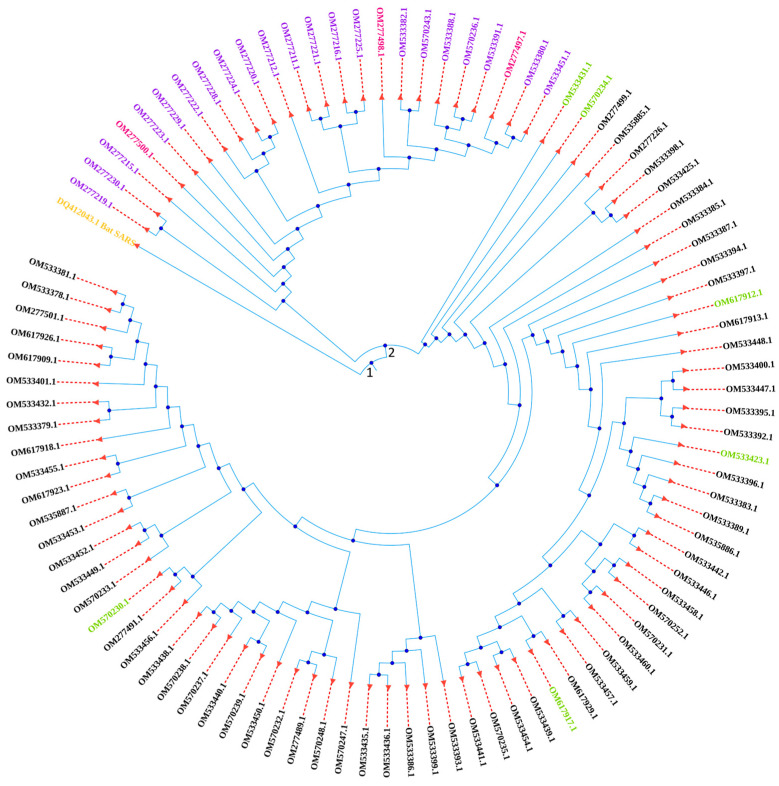
Phylogenetic tree of the whole genome of the 96 SARS-CoV-2 virus isolates and a Bat SARS coronavirus as an outgroup.

Figure 6 exhibits the phylogenetic tree of the whole genome sequence (30 Kbp in length) of 96 SARS-CoV-2 viruses of this study and a Bat SARS coronavirus as an outgroup (collected from the GenBank, NCBI), which depicts their evolutionary history. At first, the Bat SARS coronavirus and the 96 viruses split at node 1. From the second node, the 24 Delta variants and all the 72 Omicron variants branched off. The tree shows that OM277219.1 (Clade 21A), OM277230.1 (Clade 21A), and OM277215.1 (Clade 21A) are the most ancestral Delta variants and then other Delta variants (21A and 21J) emerged. Among the three variants from 21J clade of Delta, OM277500.1 emerged before the other two. On the other side, OM533431.1 and OM570234.1 variants of clade 20A emerged before the variants of clade 20B. The tree shows that among the six variants of clade 20A, two of them are close in the evolutionary relationship and the others emerged at various points in time.

## 3. Discussion

The study aimed to identify the variants of the SARS-CoV-2 virus isolates in Bangladesh (within the time frame of March 2021 to February 2022) and to compare the mutational patterns and preferences among these variants, along with their phylogenetic analysis.

In this study, the frequency of female COVID-19-positive patients (51.04%) was higher than the male patients (48.96%), which is consistent with the finding of our previous study [19]. However, there was a difference in the proportion of males and females in different divisions of Bangladesh (Supplementary Figure S1). Regarding comorbidity, the numbers of patients with asthma, diabetes mellitus, hypertension, cardiovascular disease, chronic kidney disease, and other comorbidities were 9, 31, 25, 3, 9, and 11, respectively, whereas 8 of them did not have any comorbidity (Table 1). Interestingly, it was observed that among the 18 reinfected COVID-19-positive patients, 16 (88.89% of the reinfected patients) had comorbidities, and among these 16 patients, 5, 1, and 6 of them had asthma, bronchitis, and diabetes mellitus, respectively. Supporting the tendency of co-occurrence of reinfection and comorbidities, it was reported that immune response is suppressed in respiratory disease conditions and repeated infection occurs in diabetic patients, due to the altered immune response [20,21,22]. Interestingly, the analysis revealed that even though they were vaccinated with the first, second and third doses of COVID-19 vaccines, 2, 12, and 1 of the patients, respectively, were reinfected with this virus (Supplementary Figure S4). Reinfection in vaccinated COVID-19-positive patients was also reported in other studies [20].

In this study, the identified frequencies of Delta and Omicron variants of the SARS-CoV-2 virus were 24 and 72, respectively (Table 2). In mid-2021, Delta was the dominant variant, and in late-2021 and at the beginning of 2022, Omicron replaced Delta and became the dominant one globally [23], which was also observed in this study. The clades of the Delta and Omicron variants were 21A, as well as 2J, and 20A, as well as 20B, respectively, and their frequencies were 21, as well as 3, and 6 as well as 66, respectively (Table 2). In our window of study period, clade 20B was the predominant variant in all the divisions of Bangladesh (Figure 1).

This study revealed that in these four clades, the frequencies of substitutions were much higher than the frequencies of deletions and insertions (Supplementary Figure S5). Moreover, deletions were observed at around 5% in clades 21A, and 21J, whereas in clades 20A and 20B, the percentages were more than 10%.

Nucleotide substitutions’ evaluation at variant level showed that 19 substitutions (C10029T, C27752T, T26767C, T27638C, A23403G, G15451A, G210T, C241T, C25469T, C14408T, G28881T, G28916T, C27874T, C16466T, C19220T, G4181T, C6402T, A11201G, A11332G) were observed in all of the Delta variants and 7 substitutions (C3037T, C14408T, A18163G, C23525T, T23599G, C23604A, A24424) were present in all the Omicron variants (Table 3), whereas among these mutations, A23403G, C241T, C14408T, C23604A, C25469T and C3037T were reported to be frequent in other studies [24,25].

Analysis of the nucleotide base changing pattern in Delta and Omicron showed that C > T and A > G substitutions were observed at high frequency (Table 4). Contrasting observation of preference was noted for G > A, C > A, C > G, A > T, T > G, A > C, and G > T. Deamination is believed to play a role in C > T and A > G transition mutations in the SARS-CoV-2 viral genome [26,27]. Moreover, in both of the variants, the percentages of transition mutations were almost 2-fold higher than the transversions’ (Supplementary Table S4).

A comparison of the amino acid substitutions in Delta and Omicron demonstrated that 14 (ORF1a:A1306S, ORF1a:T3255I, ORF1a:T3646A, ORF1a:P2046L, ORF1b:A1918V, ORF1b:G662S, ORF1b:P1000L, S:D614G, ORF3a:S26L, M:I82T, ORF7a:T120I, ORF7b:T40I, N:G215C, and N:R203M) were observed in all of the Delta, whereas 6 (ORF1b:I1566V, ORF1b:P314L, S:P681H, S:Q954H, S:H655Y, and S:N679K) were observed in all of the Omicron (Table 5). Statistical analysis showed that ORF1a:P2046L, ORF1a:T3646A, ORF1b:A1918V, ORF1b:G662S, S:E156G, S:G446V, S:L452R, S:T19R, ORF3a:S26L, M:I82T, ORF7b:T40I, ORF8:S54*, and N:M1X, were preferred by Delta and ORF1b:P314L, S:A67V, S:G142D, and S:N969K were preferred by Omicron (*p* values < 0.05, Supplementary Table S6).

Correlation and co-occurrence of amino acid changes across different variants indicate that these may give advantages to viral survival [28]. Moreover, the interplay of these correlated mutations among structural proteins may have an impact on the pathogenicity of the virus and vaccine efficacy [29]. The S:L452R contributes to enhancing viral fusogenicity, viral infectivity, immune escape, and reduced neutralization by antibodies [17]. In this study, L452R mutation was observed in 16 (66.67%) of the 24 Delta variants, while its frequency in the Omicron variant was 1 (1.39%) (Table 5). Therefore, this finding suggests that this mutation may have significance in viral evolution and variant selection [17]. Moreover, S:D614G, N:G204R, and N:R203K are implicated in influencing the infectivity and virulence of the virus [16]. In our study, these were in high frequency in Omicron, whereas among these three, in Delta, S:D614G was observed in all of the variants, N:G204R was not reported, and N:203 was substituted to M. The S:P618R is believed to be involved in the enhancement of SARS-CoV-2 virus transmissibility and in our study, this was observed at a high frequency in Delta, but in Omicron P618 was substituted with H (Table 5).

Some similarities and dissimilarities were observed in the patterns of deletions at the genomic sequence level in Delta and Omicron (Supplementary Table S8). Twenty variants of clade 21A followed the same deletion pattern and the other four variants of 21A and 21J had different deletion patterns. Deletion patterns of the 6 variants of clade 20A showed diversity, while 27, 21, and 14 variants of clade 20B followed three distinct deletion patterns. One deletion mutation (S:Y144-, Supplementary Table S9) was observed in these four clades and was reported to be associated with the decreased efficacy of vaccine [30].

A comparison of substitutions and deletions in Delta and Omicron showed that high-frequency substitutions were observed in ORF1a, ORF1b, S, N, and ORF7a protein (Figure 3a) of Delta, whereas in Omicron, highly substituted proteins were the S, ORF1a, ORF1b, N, ORF9b and M protein (Figure 3b). Although both deletions and substitutions were reported in the S and ORF8 protein, Delta preferred ORF8 over S for deletion, while for substitution its preference was the opposite (Figure 3a). High-frequency deletion was observed in the S, ORF1a, N, and ORF9b proteins of Omicron. The number of substitutions was higher than the number of deletions in all the proteins except for ORF8, and ORF9b proteins of Delta, and Omicron, respectively, in which deletions were preferred over substitutions (Figure 3). A study claimed that amino acid mutations occur most often in ORF1a, ORF1b, S, N, and ORF8 proteins, which influence viral infectivity and virulence [16].

The percent column chart of the substituted amino acids in different proteins reflects the differential preferences of Delta and Omicron in selecting amino acids for substitution (Figure 4a). For substitution in the spike protein, both Delta and Omicron preferred Thr, Asp, and Gly, though Omicron showed a higher preference for Asn, Ser, and Gln, and Delta showed a lower preference for them. A similar preference for Thr and Pro was observed in the ORF1a protein of Omicron and Delta, whereas Ala, Val, Leu, and Ser were differentially preferred for being substituted. For substitution in ORF1b protein, Omicron and Delta showed first preference for Pro, but they differed in preference for Ala, Gly, Val, and Ile. Similarly, in N protein, Omicron and Delta showed similar preference for Gly and Arg, whereas their preference for Asp, Met, Pro, and Ser differed.

The percent column in Figure 4b shows the similarities and dissimilarities in the selection of mutant amino acids for substitution by Omicron and Delta. In the S protein, Omicron and Delta preferred Lys, Arg, and His as mutant amino acids, whereas Omicron also showed a preference for Tyr, Phe, Asp, Asn, Ser, and Ala, but Delta did not. In ORF1a protein of Delta and Omicron, a high preference for Ile was observed, whereas they showed a differential preference for mutant Leu, Ser, Ala, Phe, His, and Arg. In ORF1b protein, both Delta and Omicron showed a high preference for Leu and Val, whereas Delta also preferred Phe and Ser, mutant amino acids, but Omicron did not prefer them. In protein N, Delta and Omicron showed a differential preference for Arg, Leu, Lys, Cys, Met, Gly, and Tyr mutant amino acids. These differential preferences for substituting and mutant amino acids by the Omicron and Delta highlight their importance in viral evolution and adaptability, which implies the requirement of their comprehensive analysis with big data from around the globe [31]. 

Analysis of deleted amino acids in Omicron and Delta unveiled their varied preferences for amino acids in deletions (Figure 5). In the ORF8 protein, Delta exclusively selected Asp and Phe, whereas Omicron had a very low-frequency deletion of Asp and Phe in this protein. Although in the S protein, Delta preferably deleted Phe and Arg the most, it also deleted Tyr, Glu, Val and His, but at a low frequency, whereas Omicron mostly preferred Tyr, Val, and Pro for deletion and also deleted His, Ala, and Leu, but at a lower frequency. Omicron had a high-frequency deletion in ORF1a, ORF9b, and N protein, and in ORF1a it preferably deleted Phe, Ser, and Gly the most, and Leu at a lower frequency. For deletion in the ORF9b protein, Omicron preferred Ala, Asn, and Val, whereas in the N protein it selectively deleted Arg, Glu, and Ser.

Mutational analysis showed that all the insertions were frame preserving and, compared to other clades, a higher number of insertions were observed in clade 20B. An insertion (22206:GCCAGAAGA) that added an extra three amino acids (EPE) at the trimer interface of the N-terminal domain of the S1 subunit of spike glycoprotein was observed in 11 variants of this clade (Table 6), which might render structural changes in the S protein’s S1 subunit, which is mainly involved in attachment and interaction with the host receptor [10]. Moreover, these membrane receptors and proteases through which SARS-CoV-2 attains entry into the host cell are found in most of the organs of the human body, including the lung, brain, kidney, liver, gastrointestinal tract, and spleen [32,33,34,35,36,37]. Therefore, the variant S protein of Omicron and Delta can possibly interact differently with its receptor proteins throughout the human body system. Moreover, the insertion of 12bp in length (2902:GTGTTGTGGCAG) added four amino acids (“VLWQ”) in the nsp3a protein’s N terminal domain and this protein is reported to be involved in several pathogenicity and immune evasion pathways [14]. No insertion mutation was found in the three variants of clade 21J.

Like other viruses, the SARS-CoV-2 virus depends on the host’s cellular components and pathways for its successful replication cycle and host immune evasion [38]. During this process, the virus can cause several abnormalities and impairment in the host’s biological system, i.e., perturbation of epigenetic regulations, and metabolic homeostasis, leading to cellular destruction, and disruption of host immune response, furthermore facilitating the pathogenesis and progression of the disease [2]. It was observed that the prevalence and severity of post-COVID-19 condition, interchangeably termed “long-COVID”, vary with the infecting variants of SARS-CoV-2, i.e., Omicron and Delta [39]. Moreover, reinfection with Omicron is associated with more severe long-term symptoms than first-time infection with Omicron [40,41]. The difference in mutation patterns in different genes, i.e., S gene, of the variants may contribute to the development and variation of long-COVID prevalence and symptoms [42]. Therefore, to understand viral behaviour and strategies in the human body system, to track viral evolution, and to anticipate the emergence of new variants of concern, comparison of the mutation profile is also crucial for Bangladesh.

The phylogenetic analysis showed that, being split at node 1 from the Bat SARS coronavirus, the Delta and Omicron variants branched off at node 2 (Figure 6). The tree also shows that three variants from clade 21A and 2 variants from 20A emerged before the emergence of variants from clade 21J and 20B, respectively. Through the acquisition of substitution, deletion and insertion mutations, the variants emerged with a new genetic makeup.

## 4. Materials and Methods

### 4.1. Study Subjects

The cross-sectional descriptive study was reviewed and approved by the Institutional Review Board (IRB) of Bangladesh Medical University (BMU) under the declaration of Helsinki ethical principles (No. 3506, date 28 June 2021). This study was conducted in the Genomics Research Laboratory of the Department of Anatomy, Bangladesh Medical University, Bangladesh. The time frame of this study was from March 2021 to February 2022. Suspected COVID-19-positive patients were informed about the purpose of this research work and asked for consent. The socio-demographic and comorbidity data of the confirmed COVID-19-positive patients were collected through a questionnaire administered to the patients.

### 4.2. Viral Genomic RNA Extraction, Sequencing, and NGS Data Analysis

The genomic RNA of the SARS-CoV-2 virus was extracted from the nasopharyngeal swab specimens of the study participants, genomic RNA was sequenced, and NGS data analysis was performed following our previous study on COVID-19 [43]. The final sequence data were uploaded to the GenBank Database. The accession numbers of the 96 identified SARS-CoV-2 viral genome sequences of this study are provided in the supplemental Excel datasheet named “Accession Numbers of 96 Seq_Data”.

### 4.3. Statistical Analysis and Figure Generation

For statistical analysis of the mutational data, R studio and Microsoft Excel were used. To construct the figures, online platforms named SRplot (SRplot—Science and Research online plot, the updated version (last used in 2025)) [44], and jvenn (the current version (last assessed on 2025), jvenn: an interactive Venn diagram viewer) [45], and Microsoft Excel were used. For phylogenetic analysis of the 96 viral genomes, Unipro UGENE version 51.0 (Unipro UGENE—Integrated Bioinformatics Tools) was used [46,47]. For multiple sequence alignment (MSA), the Clustal Omega algorithm was selected. PhyML maximum likelihood method was employed for the construction of the phylogenetic tree in which a branch support SH-like fast likelihood-based method was selected for having accurate and fast output [48]. To adjust and customize the structure of the tree, and to label colours, styles, and fonts of the tree, iTOL version 7.0 was used [49,50].

## 5. Conclusions

In conclusion, the findings of the study point out the variations of Delta and Omicron of SARS-CoV-2 virus, from the perspective of mutation pattern, wild type as well as mutant amino acid preferences for substitutions and deletions, and preferences in mutation site as well, which might help in understanding the basis of viral mutational strategy in Bangladesh within this window of the time-period of our study during the pandemic situation. However, we have a limitation in sample size, which is 96 viral isolates in this study; the differential mutational profile of Omicron and Delta would be better understood if the sample size were in the thousands.

## Figures and Tables

**Table 1:** Frequency of socio-demographic and comorbidity data of the COVID-19-positive patients.

Socio-Demography and Comorbidity		COVID-19-Positive Patients, *n* = 96
		*n* (%)
Gender	Male	47 (48.96)
	Female	49 (51.04)
Comorbidities	Asthma	9 (9.38)
	Diabetes Mellitus	31 (32.29)
	Hypertension	25 (26.04)
	Cardiovascular disease	3 (3.13)
	Chronic kidney disease	9 (9.38)
	Others	11 (11.46)
	No comorbidity	8 (8.33)
Vaccination state	First dose vaccinated	17 (17.71)
	Second dose vaccinated	47 (48.96)
	Third dose vaccinated	7 (7.29)
	Non-vaccinated	25 (26.04)
History of long-distance travelling	Yes	12 (12.50)
	No	84 (87.50)
Family history of COVID-19 Infection	Yes	31 (32.29)
	No	29 (30.21)
	Patient could not confirm	36 (37.50)
Re-infected with SARS-CoV-2	Yes	18 (18.75)
	No	78 (81.25)

**Table 2:** Frequency of identified variants and clades of the 96 SARS-CoV-2 virus isolates.

Variant	Number	Percentage (%)	Clade	Number	Percentage (%)
Delta	24	25	21A	21	21.88
			21J	3	3.13
Omicron	72	75	20A	6	6.25
			20B	66	68.75

**Table 3:** Frequency of nucleotide substitution mutations in the Delta and Omicron variants.

Delta		Omicron			
Substitution Mutation	Number of Mutation, *n* (%)	Substitution Mutation	Number of Mutation, *n* (%)	Substitution Mutation	Number of Mutation, *n* (%)
C10029T	24 (100.00)	C3037T	72 (100.00)	C24503T	43 (59.72)
C27752T	24 (100.00)	C14408T	72 (100.00)	A2832G	40 (55.56)
T26767C	24 (100.00)	A18163G	72 (100.00)	C22686T	40 (55.56)
T27638C	24 (100.00)	C23525T	72 (100.00)	C22674T	38 (52.78)
A23403G	24 (100.00)	T23599G	72 (100.00)	T22679C	38 (52.78)
G15451A	24 (100.00)	C23604A	72 (100.00)	T22882G	37 (51.39)
G210T	24 (100.00)	A24424T	72 (100.00)	G22813T	34 (47.22)
C241T	24 (100.00)	A23403G	71 (98.61)	A22688G	27 (37.50)
C25469T	24 (100.00)	C25584T	71 (98.61)	T670G	26 (36.11)
C14408T	24 (100.00)	G26709A	71 (98.61)	G22775A	26 (36.11)
G28881T	24 (100.00)	A27259C	71 (98.61)	A22786C	26 (36.11)
G28916T	24 (100.00)	C26270T	70 (97.22)	C26060T	26 (36.11)
C27874T	24 (100.00)	C10029T	69 (95.83)	G4184A	25 (34.72)
C16466T	24 (100.00)	G22578A	68 (94.44)	C4321T	25 (34.72)
C19220T	24 (100.00)	C23854A	68 (94.44)	C9534T	25 (34.72)
G4181T	24 (100.00)	T24469A	68 (94.44)	C9866T	25 (34.72)
C6402T	24 (100.00)	C25000T	68 (94.44)	C12880T	25 (34.72)
A11201G	24 (100.00)	A28271T	68 (94.44)	C15714T	25 (34.72)
A11332G	24 (100.00)	C28311T	68 (94.44)	C17410T	25 (34.72)
C8986T	23 (95.83)	G23948T	67 (93.06)	C19955T	25 (34.72)
C21618G	23 (95.83)	C26577G	66 (91.67)	A20055G	25 (34.72)
G9053T	22 (91.67)	G28881A	66 (91.67)	G21987A	25 (34.72)
C23604G	22 (91.67)	G28882A	66 (91.67)	C26858T	25 (34.72)
C3037T	22 (91.67)	G28883C	66 (91.67)	G27382C	25 (34.72)
A28461G	20 (83.33)	C10449A	65 (90.28)	T27384C	25 (34.72)
C14407T	19 (79.17)	C241T	63 (87.50)	C2790T	24 (33.33)
C28054G	19 (79.17)	C27807T	62 (86.11)	C21618T	24 (33.33)
G29402T	19 (79.17)	C21846T	47 (65.28)	G22898A	24 (33.33)
T29014C	18 (75.00)	G8393A	46 (63.89)	G23048A	24 (33.33)
G19999T	17 (70.83)	T13195C	46 (63.89)	A27383T	24 (33.33)
T22917G	16 (66.67)	A23040G	46 (63.89)	A29510C	24 (33.33)
C22995A	15 (62.50)	T23075C	46 (63.89)	C9344T	23 (31.94)
G22899T	15 (62.50)	C24130A	46 (63.89)	A9424G	23 (31.94)
C7124T	15 (62.50)	T5386G	45 (62.50)	G10447A	23 (31.94)
C21846T	13 (54.17)	A11537G	45 (62.50)	T22200G	23 (31.94)
A23064C	12 (50.00)	C21762T	45 (62.50)	C2470T	21 (29.17)
G29742T	12 (50.00)	A23063T	45 (62.50)	G22599A	21 (29.17)
G29688T	11 (45.83)	C15240T	44 (61.11)	T16342C	19 (26.39)
G24410A	7 (29.17)	G22992A	44 (61.11)	A26530G	18 (25.00)
A2903G	6 (25.00)	A23055G	44 (61.11)	C26522T	14 (19.44)
C20148T	4 (16.67)	C22995A	43 (59.72)	C10198T	13 (18.06)
G23012C	3 (12.50)	A23013C	43 (59.72)	T22673C	10 (13.89)
		C23202A	43 (59.72)		

**Table 4:** Nucleotide base changing pattern in the whole genome of Delta and Omicron.

Delta			Omicron		
Nucleotide Base Change	Number	Percentage (%)	Nucleotide Base Change	Number	Percentage (%)
A > G	114	12.43	A > G	424	11.17
A > C	14	1.53	A > C	168	4.43
A > T	1	0.11	A > T	212	5.58
C > T	360	39.26	C > T	1351	35.59
C > G	64	6.98	C > G	70	1.84
C > A	19	2.07	C > A	340	8.96
G > T	211	23.01	G > T	119	3.13
G > A	41	4.47	G > A	545	14.36
G > C	6	0.65	G > C	93	2.45
T > C	66	7.20	T > C	200	5.27
T > G	17	1.85	T > G	204	5.37
T > A	4	0.44	T > A	70	1.84
Total	917	100.00	Total	3796	100.00

**Table 5:** Frequency of amino acid substitution mutations in different proteins of Delta and Omicron.

Delta			Omicron		
Protein	Amino Acid Mutation	Number of Mutation, *n* (%)	Protein	Amino Acid Mutation	Number of Mutation, *n*(%)
ORF1a	A1306S	24 (100.00)	ORF1a	T3255I	69 (95.83)
	T3255I	24 (100.00)		P3395H	65 (90.28)
	T3646A	24 (100.00)		A2710T	46 (63.89)
	P2046L	24 (100.00)		S2083I	46 (63.89)
	V2930L	22 (91.67)		I3758V	45 (62.50)
	P2287S	15 (62.50)		L3674F	44 (61.11)
	I880V	6 (25.00)		K856R	40 (55.56)
	H417Y	2 (8.33)		S135R	26 (36.11)
	I3618V	2 (8.33)		T3090I	25 (34.72)
	P309L	2 (8.33)		G1307S	25 (34.72)
	P4220S	1 (4.17)		L3201F	25 (34.72)
	Q1332H	1 (4.17)		T842I	24 (33.33)
	Q1784H	1 (4.17)		L3027F	23 (31.94)
	S1515F	1 (4.17)		V1887I	4 (5.56)
	S2631F	1 (4.17)		T1822I	3 (4.17)
	F536V	1 (4.17)		P2046L	3 (4.17)
	K2497N	1 (4.17)		T3646A	2 (2.78)
	C270F	1 (4.17)		H417Y	1 (1.39)
	T2306I	1 (4.17)		I880V	1 (1.39)
	T746I	1 (4.17)		L3606F	1 (1.39)
	K3353R	1 (4.17)	ORF1b	I1566V	72 (100.00)
	L3606F	1 (4.17)		P314L	72 (100.00)
	L628P	1 (4.17)		R1315C	25 (34.72)
	M3761I	1 (4.17)		T2163I	25 (34.72)
	P1158L	1 (4.17)		S959P	19 (26.39)
	P1497L	1 (4.17)		G1093S	3 (4.17)
	P1977L	1 (4.17)		G662S	2 (2.78)
ORF1b	A1918V	24 (100.00)		A1918V	2 (2.78)
	G662S	24 (100.00)	S	P681H	72 (100.00)
	P1000L	24 (100.00)		Q954H	72 (100.00)
	P314F	19 (79.17)		H655Y	72 (100.00)
	V2178F	17 (70.83)		N679K	72 (100.00)
	P314L	5 (20.83)		G142D	71 (98.61)
	A2131V	2 (8.33)		D614G	71 (98.61)
	H1087Y	2 (8.33)		N969K	68 (94.44)
	I2158V	2 (8.33)		G339D	68 (94.44)
	Q2247H	1 (4.17)		N764K	68 (94.44)
	Q348H	1 (4.17)		D796Y	67 (93.06)
	S2379L	1 (4.17)		T95I	47 (65.28)
	T1555I	1 (4.17)		Q493R	46 (63.89)
	D131Y	1 (4.17)		Y505H	46 (63.89)
	G2610S	1 (4.17)		N856K	46 (63.89)
	T2165M	1 (4.17)		A67V	45 (62.50)
	V464F	1 (4.17)		N501Y	45 (62.50)
	M1596I	1 (4.17)		Q498R	44 (61.11)
S	D614G	24 (100.00)		S477N	44 (61.11)
	T19R	23 (95.83)		T478K	43 (59.72)
	P681R	22 (91.67)		T547K	43 (59.72)
	E156G	21 (87.5)		E484A	43 (59.72)
	L452R	16 (66.67)		L981F	43 (59.72)
	G446V	15 (62.5)		S375F	40 (55.56)
	T478K	15 (62.5)		S373P	38 (52.78)
	T95I	13 (54.17)		N440K	37 (51.39)
	N501T	12 (50)		K417N	34 (47.22)
	D950N	7 (29.17)		S371F	28 (38.89)
	E484Q	3 (12.5)		T376A	27 (37.50)
	G142D	2 (8.33)		D405N	26 (36.11)
	R158G	2 (8.33)		R408S	26 (36.11)
	A67V	1 (4.17)		G446S	24 (33.33)
	N969K	1 (4.17)		G496S	24 (33.33)
	D80H	1 (4.17)		T19I	24 (33.33)
	S929T	1 (4.17)		L24S	24 (33.33)
ORF3a	S26L	24 (100.00)		V213G	23 (31.94)
	V202L	2 (8.33)		R346K	21 (29.17)
	S74F	1 (4.17)		N211I	14 (19.44)
	T221K	1 (4.17)		S371L	10 (13.89)
	Y145H	1 (4.17)		A701V	4 (5.56)
	G49V	1 (4.17)		G798D	4 (5.56)
E	S68Y	1 (4.17)		V1264L	3 (4.17)
				F643L	3 (4.17)
				G446V	1 (1.39)
M	I82T	24 (100.00)		T19R	1 (1.39)
ORF7a	T120I	24 (100.00)		L452R	1 (1.39)
	V82A	22 (91.67)		N501T	1 (1.39)
	V82S	2 (8.33)		E156G	1 (1.39)
	P34L	1 (4.17)	ORF3a	T223I	26 (36.11)
ORF7b	T40I	24 (100.00)		D155Y	3 (4.17)
				S26L	1 (1.39)
			E	T9I	70 (97.22)
ORF8	S54*	19 (79.17)	M	A63T	71 (98.61)
	P38L	2 (8.33)		Q19E	66 (91.67)
	C83Y	1 (4.17)		D3G	18 (25.00)
	K68*	1 (4.17)		I82T	1 (1.39)
	L7*	1 (4.17)	ORF6	D61L	24 (33.33)
	L98I	1 (4.17)	ORF7b	T40I	5 (6.94)
ORF9b	T60A	20 (83.33)	ORF8	S54*	3 (4.17)
	G38D	2 (8.33)	ORF9b	E27V	68 (94.44)
	R32L	1 (4.17)		P10S	68 (94.44)
N	G215C	24 (100.00)	N	P13L	68 (94.44)
	R203M	24 (100.00)		G204R	66 (91.67)
	M1X	22 (91.67)		R203K	66 (91.67)
	D63G	20 (83.33)		S413R	24 (33.33)
	D377Y	19 (79.17)		M1X	3 (4.17)
	A359S	1 (4.17)		G215S	1 (1.39)

ORF 1a, 1b, 3a, 6, 7a, 7b, 8, and 9b = Open Reading Frame 1a, 1b, 3a, 6, 7a, 7b, 8, and 9b protein, S = Spike protein, E = Envelope protein, M = Membrane protein, N = Nucleocapsid protein.

**Table 6:** Insertion mutations and their genomic locations in different variants and clades of the SARS-CoV-2 virus.

Variant	Clade	Genomic Position: Inserted Sequence	Number	Insertion Length (bp)	Mapped Genomic Region
Delta	21A	2902:GTGTTGTGGCAG	1	12	*ORF1a* (NSP3)
Omicron	20B	22206:GCCAGAAGA	11	9	*S* (S1 subunit)
	20A	75:AAAC	1	4	5′-UTR
		76:AAA	1	3	5′-UTR

5′-UTR = 5′-untranslated region.

## Data Availability

All the findings of the study are presented in this article and other findings are provided as the Supplementary Materials. The raw data, accession numbers of the 96 SARS-CoV-2 virus genomic sequences, and the Supplementary Information were uploaded to figshare and are available for downloading at https://doi.org/10.6084/m9.figshare.28912049, accessed on 26 May 2025.

## Supplementary Materials

The following supporting information was uploaded to figshare and can be downloaded from https://doi.org/10.6084/m9.figshare.28912049 (accessed on 1 May 2025). Supplementary Materials include Supplementary Tables and Figures alongside an Excel datasheet of unique mutations in different clades.
